# Genetic risk, parental history, and suicide attempts in a diverse sample of US adolescents

**DOI:** 10.3389/fpsyt.2022.941772

**Published:** 2022-09-14

**Authors:** Ran Barzilay, Elina Visoki, Laura M. Schultz, Varun Warrier, Nikolaos P. Daskalakis, Laura Almasy

**Affiliations:** ^1^Department of Child and Adolescent Psychiatry and Behavioral Science, Children's Hospital of Philadelphia (CHOP), Philadelphia, PA, United States; ^2^Lifespan Brain Institute of CHOP and Penn Medicine, Philadelphia, PA, United States; ^3^Department of Psychiatry, Perelman School of Medicine, University of Pennsylvania, Philadelphia, PA, United States; ^4^Department of Biomedical and Health Informatics, Children's Hospital of Philadelphia (CHOP), Philadelphia, PA, United States; ^5^Department of Psychiatry, University of Cambridge, Cambridge, United Kingdom; ^6^Department of Psychiatry, McLean Hospital and Harvard Medical School, Belmont, MA, United States; ^7^Stanley Center for Psychiatric Research, Broad Institute of MIT and Harvard, Cambridge, MA, United States; ^8^Department of Genetics, Perelman School of Medicine, University of Pennsylvania, Philadelphia, PA, United States

**Keywords:** suicide attempt, genetics, polygenic risk prediction, family history, adolescents, child adolescent psychiatry

## Abstract

**Background:**

Adolescent suicide is a major health problem in the US marked by a recent increase in risk of suicidal behavior among Black/African American youth. While genetic factors partly account for familial transmission of suicidal behavior, it is not clear whether polygenic risk scores of suicide attempt can contribute to suicide risk classification.

**Objectives:**

To evaluate the contribution of a polygenic risk score for suicide attempt (PRS-SA) in explaining variance in suicide attempt by early adolescence.

**Methods:**

We studied *N* = 5,214 non-related youth of African and European genetic ancestry from the Adolescent Brain Cognitive Development (ABCD) Study (ages 8.9–13.8 years) who were evaluated between 2016 and 2021. Regression models tested associations between PRS-SA and parental history of suicide attempt/death with youth-reported suicide attempt. Covariates included age and sex.

**Results:**

Over three waves of assessments, 182 youth (3.5%) reported a past suicide attempt, with Black youth reporting significantly more suicide attempts than their White counterparts (6.1 vs. 2.8%, *p* < 0.001). PRS-SA was associated with suicide attempt [odds ratio (OR) = 1.3, 95% confidence interval (CI) 1.1–1.5, *p* = 0.001]. Parental history of suicide attempt/death was also associated with youth suicide attempt (OR = 3.1, 95% CI, 2.0–4.7, *p* < 0.001). PRS-SA remained significantly associated with suicide attempt even when accounting for parental history (OR = 1.29, 95% CI = 1.1–1.5, *p* = 0.002). In European ancestry youth (*n* = 4,128), inclusion of PRS-SA in models containing parental history explained more variance in suicide attempt compared to models that included only parental history (Δ*R*^2^ = 0.7%, *p* = 0.009).

**Conclusions:**

Findings suggest that PRS-SA may be useful for youth suicide risk classification in addition to established risk factors.

## Introduction

Suicide is the second leading cause of death in US adolescents (1). The rising rates of suicide among Black or African American youth are especially concerning (2). Suicide attempt is a complex behavior influenced by multiple risk factors including preexisting psychopathology, interpersonal stressors, socioeconomic, and genetic factors (3). Clinicians often use parental history of suicide attempt/death to estimate suicide risk (4). The potential of using polygenic scores of psychiatric phenotypes to assess genetic suicide risk is uncertain (5). It is not known whether a polygenic score of suicide attempts (PRS-SA) can contribute to suicide risk classification or whether PRS-SA adds useful information beyond the commonly used risk assessment based on parental history.

The Adolescent Brain Cognitive Development (ABCD) Study follows diverse genotyped US youth from ages 9–10 into adolescence (6). The study collects data on parental history of psychiatric conditions (7), including suicide attempt/death. Participants are evaluated annually for history of suicide attempts, and endorsement of suicide attempts in Black participants is significantly higher (8). Here, we aimed to evaluate the contribution of PRS-SA to explaining variance in self-reported suicide attempt by early adolescence and to determine the additive effect of this score over and above parental history of suicide attempt/death.

## Methods

### Participants

We included *N* = 5,214 non-related ABCD Study participants of African and European genetic ancestry who had data on parental history of suicide attempt or death (*n* = 302 missing such data were excluded from analyses). From each family, only the oldest sibling was selected for this study (*n* = 1,002 siblings were excluded: Supplementary Figure 1). We imputed age at clinical assessment for the 21 participants (0.4%) included in our analysis who did not complete the last ABCD Study assessment.

Of the total sample, *n* = 1,086 were classified as having African genetic ancestry [of whom 988 (97.1%) were parent reported as being Black and 71 (6.6%) were parent reported being Hispanic]; and *n* = 4,128 had European genetic ancestry [of whom 4,093 (99.2%) were parent reported as being White and 123 (3%) were parent reported as being Hispanic]. The ABCD Study^®^ protocol was approved by the University of California, San Diego Institutional Review Board (IRB) and was exempted from a full review by University of Pennsylvania IRB.

### Genotyping, quality control, and imputation

ABCD genotype data were obtained from saliva samples using the Affymetrix NIDA SmokeScreen array (NDA #2573, fix release 2.0.1). We used PLINK 1.9 (9) to remove single nucleotide polymorphisms (SNPs) with >5% missingness, samples with more than 10% missingness, and samples with a genotyped sex that did not match the reported sex phenotype. Then, we compared SNP frequencies against the 1,000 Genomes ALL reference panel (10). This fixed strand reversals and improper Ref/Alt assignments and also removed palindromic A/T and C/G SNPs with minor allele frequency (MAF) >0.4, SNPs with alleles that did not match the reference panel, SNPs with allele frequencies differing by more than 0.2 from the reference, and SNPs not present in the reference panel. The pre-imputation QC process yielded a genomic dataset comprised of 485,329 variants and 10,318 individuals.

Genotypes were phased (Eagle v.2.4) and imputed by chromosome to the 1,000 Genomes Other/Mixed GRCh37/hg19 reference panel (Phase 3 v.5) using Minimac 4 *via* the Michigan Imputation Server (11). All post-imputation QC was run using bcftools (12). Only polymorphic sites with imputation quality *R*^2^ ≥ 0.7 and MAF ≥ 0.01 were included in the final PLINK 1.9 hard-call post-imputation dataset comprised of 9,768,092 variants.

Multidimensional scaling (MDS) was conducted using KING (v.2.2.4) (13) to identify the top ten ancestry components for each sample. The ancestry PCs were projected onto the 1,000 Genomes PC space, and genetic ancestry was inferred using the e1071 (14) support vector machines package in R version 4.1.0 (15). The African (*n* = 1,741) and European (*n* = 5,815) ancestry individuals eligible to be included in the present study were defined by these inferences; all other ancestry groups were excluded from further analysis. A second round of unprojected MDS was then performed within the EUR- and AFR-ancestry groups to produce ten PCs that were regressed out of the standardized PRS-SA to adjust for genetic ancestry.

## Variables

### Exposures

#### Polygenic risk score of suicide attempt

Summary statistics were obtained for a suicide attempt genome-wide association study (GWAS) meta-analysis run by the International Suicide Genetics Consortium (16). Given that this is a trans-ancestry GWAS (i.e., ~90% EUR, ~6% Asian, and ~4% admixed; 29,782 suicide attempt cases and 519,961 controls), we opted to use PRSice-2 (17) to compute PRS-SA separately for the African and European ancestry ABCD participants to allow for differing linkage disequilibrium (LD) structure in these groups. SNPs in the two target datasets were clumped to minimize LD using an *r*^2^ ≥ 0.1 threshold in sliding windows of 250 kB and then selected from the discovery GWAS for inclusion in the PRS-SA calculations based on a series of eight *P*-value thresholds, ranging from 0.0001 to 1 (Supplementary Table 1). Raw PRS-SA was computed at each *P*-value threshold by summing the effect alleles weighted by the log-odds ratio estimated by the discovery GWAS. The two ancestry-specific sets of PRS-SA were then z-scored and corrected for population stratification by regressing out 10 within-ancestry PCs at each *P*-value threshold, yielding eight PRS-SA per study participant.

#### Parental history of suicide attempt

Parental history was evaluated using parent reports on parents' suicide attempt/death (variable: “famhx_ss_momdad_scd_p”) in the first assessment of the ABCD study.

### Outcome measure

The ABCD Study clinical assessment was based on the Kiddie Schedule for Affective Disorders and Schizophrenia 5 (KSADS-5) and included detailed questions on suicidal thoughts and behavior (18, 19). The participants were specifically asked about history of suicide attempt (“was there ever a time when you did something to try to kill yourself and actually made a suicide attempt?”), including aborted or interrupted attempts [“did you start to do something to end your life, but either stopped yourself or were interrupted by someone else (for example, you were about to take pills or had a gun ready, or were about to jump or hang yourself, but either stopped yourself or were stopped by someone else?)”].

The participants who endorsed any of the above questions at least one time in any of the three first ABCD Study assessments were considered as suicide attempters. The participants who denied history of suicide attempt in all three assessments were considered controls. All other participants [*n* = 1,038] were excluded from the analysis.

### Statistical analyses

Analyses were conducted from January-March 2022 using ABCD Study data release 4.0. We used R version 4.1.0. for data analyses. Data preprocessing and analysis are detailed at https://github.com/barzilab1/ABCD_SA_genetics_FH.

Mean [standard deviation (*SD*)] and frequency (%) were reported for descriptive purposes. Univariate comparisons were made using *t*-test or chi-square tests, as appropriate. We used two-tailed tests for all statistical models.

We estimated binary logistic regression models with suicide attempt as the dependent variable and PRS-SA as the independent variable, co-varying for age and sex. To allow inclusion of the participants from diverse ancestries in our analyses, we estimated models stratified by ancestry and then meta-analyzed the results. We determined the optimal GWAS *P*-value threshold for the PRS-SA based on the highest odds ratio and lowest *P*-value of PRS-SA in association with suicide attempt in the meta-analyzed results.

To explore the additive effects of PRS-SA on explaining variance in suicide attempt, estimated by Nagelkerke's *R*^2^, we estimated stratified regression models in the African and European ancestry youth with and without PRS-SA and compared the goodness of fit using the likelihood ratio test.

## Results

Among the 5,214 participants, 182 (3.5%) endorsed having made a suicide attempt at least one time in the three ABCD Study assessments. History of suicide attempt was more frequent among Black youth (66 of 1,087; 6.1%) than among their White counterparts (116 of 4,127; 2.8%, chi-square *p* < 0.001). No age or sex associations were observed. The participants who endorsed suicide attempt had more parental history of suicide attempt/death (14.8 vs. 5.5%, respectively, chi-square *p* < 0.001). Table 1 includes univariate comparisons between participants with and without history of a suicide attempt. Association of PRS-SA with suicide attempt was consistent across multiple *P*-value threshold tested, accounting for age, sex, and for ten within-ancestry genetic principal components (see Supplementary Table 2). Out of the eight GWAS *P*-value thresholds for the PRS-SA tested, the one that achieved the highest odds ratio and lowest *P*-value of PRS-SA was *p* = 0.05. We used a permutation test to validate this selection (see Supplementary Figure 2).

**Table 1:** Sample characteristics and univariate comparison between suicide attempters and controls.

	**Total sample**	**Control**	**Suicide attempt**	***P*-value**
	***N* = 5,214**	***n* = 5,032**	***n* = 182**	
Age, years, mean (SD)	12.04 (0.65)	12.04 (0.65)	12.09 (0.66)	0.325
Female sex, No. (%)	2,408 (46.2)	2,319 (46.1)	89 (48.9)	0.501
Race Black, No. (%)	1,087 (20.8)	1,021 (20.3)	66 (36.3)	<0.001
Parent suicide attempt/death, No. (%)	306 (5.9)	279 (5.5)	27 (14.8)	<0.001
Suicide attempt PRS^a^, mean (SD)	0.00 (0.95)	−0.01 (0.95)	0.21 (0.89)	0.001

a PRS after standardizing the raw PRS produced at a GWAS P-value threshold of 0.05 and then regressing out the first ten within-ancestry genetic principal components.

PRS, polygenic risk score; GWAS, Genome-wide association study.

**Figure 1:**
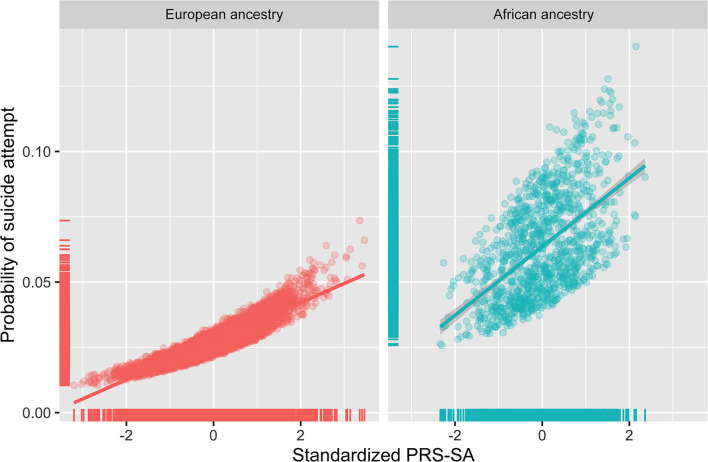
A polygenic risk score for suicide attempt (PRS-SA) and suicide attempt among youth of African and European ancestries. Scatter plots and regression lines show estimated probabilities of suicide attempt in adolescents obtained from binary logistic regression models with PRS-SA, age, and sex as independent variables. X-axis represents a PRS-SA score (after standardizing the raw PRS produced at a GWAS *P*-value threshold of 0.05 and then regressing out the first ten within-ancestry genetic ancestry principal components). Y-axis represents predicted probability of suicide attempt.

We then tested the association of PRS-SA at the *P*-value threshold of 0.05 with suicide attempt accounting for parental history of suicide attempt/death. When tested individually in separate models, both PRS-SA [odds ratio (OR) = 1.3, 95% confidence interval (CI) 1.1–1.5, *p* = 0.001] and parental history of suicide attempt/death (OR = 2.9, 95% CI = 1.9–4.4, *p* < 0.001) were significantly associated with suicide attempt in the full (meta-analyzed) sample. When included in the same model, PRS-SA remained associated with suicide attempt in a similar effect size (OR = 1.29, 95% CI = 1.1–1.5, *p* = 0.002, Table 2). Figure 1 illustrates the association between PRS-SA and the suicide attempt rate. Supplementary Table 3 includes the odds ratios obtained in the models stratified by ancestry (African or European) prior to meta-analysis. Associations of PRS-SA with suicide attempt were similar in direction in both European and African ancestries and were statistically significant in European but not in the African ancestry.

**Table 2:** Association of suicide attempt PRS, parental history of suicide attempt/death and suicide attempt in the meta-analyzed study population (*N* = 5,214).

**Predictors**	**Model 1^a^**			**Model 2^b^**			**Model 3^c^**			**Model 4^d^**		
	**OR**	**95% CI**	***P***	**OR**	**95% CI**	***P***	**OR**	**95% CI**	***P***	**OR**	**95% CI**	***P*-value**
Age	1.01	0.99–1.03	0.218	1.01	0.99–1.03	0.197	1.01	0.99–1.03	0.223	1.01	0.99–1.03	0.192
Female sex	1.11	0.82–1.50	0.488	1.11	0.82–1.50	0.494	1.12	0.83–1.52	0.454	1.12	0.83–1.52	0.454
Suicide attempt PRS^e^				1.31	1.11–1.54	0.001				1.29	1.10–1.52	0.002
Parental suicide risk^f^							2.96	1.93–4.54	<0.001	2.88	1.87–4.42	<0.001

Meta-analyzed effect sizes (odds ratio) derived from binary logistic regression models with age, sex, suicide attempt PRS, and parental history of suicide attempt/death as independent variables and self-reported suicide attempt as the dependent variable.

a Model 1 includes age and sex as independent variables.

b Model 2 includes age, sex, and suicide attempt PRS as independent variables.

c Model 3 includes age, sex, and suicide attempt parental history as independent variables.

d Model 4 includes age, sex, suicide attempt PRS, and suicide attempt parental history as independent variables.

e PRS after standardizing the raw PRS produced by PRSice-2 at a GWAS P-value threshold of 0.05 and then regressing out the first ten within-ancestry genetic ancestry principal components.

f Suicide attempt/death.

PRS, polygenic risk score; GWAS, Genome-wide association study.

Lastly, we explored the additive explanatory contribution of PRS-SA to youth suicide attempt over and above demographics (age and sex) and parental family history of suicide attempt/death (Table 3). In the European ancestry participants, the model that included PRS-SA explained 1% of the variance (Nagelkerke's *R*^2^ = 0.01), significantly more than the base model that only included age and sex that explained 0.1% of the variance (Nagelkerke's *R*^2^ = 0.001, the likelihood ratio Chi-square test, *p* = 0.004). Addition of PRS-SA to a model that included family history of parental suicide attempt/death increased the variance explained from 1.9% to 2.6% (the likelihood ratio chi-square test, *p* = 0.009). The improvement in model performance (Δ*R*^2^ = 0.7%, from 1.9% to 2.6%) obtained when adding SA-PRS was on the order of 39% of the Δ*R*^2^ obtained when adding parental history to the base model (Δ*R*^2^ = 1.8%, from 0.1% to 1.9%). In the African ancestry group, PRS-SA increased the variance explained in models explaining suicide attempt, but the differences in *R*^2^ were not statistically significant.

**Table 3:** Explained variance in youth suicide attempt derived from binary logistic models estimated separately in European and African ancestry participants.

		**European ancestry**	**African ancestry**
		***n* = 4,128**	***n* = 1,086**
	**Independent variables**	**Nagelkerke *R*^2^%**	**Nagelkerke *R*^2^%**
Model 1	Age, Sex	0.1	1.1
Model 2^a^	Age, Sex, SA PRS^d^	1.0	1.7
Model 3^b^	Age, Sex, SA parental risk^e^	1.9	2.0
Model 3^c^	Age, Sex, SA parental risk^e^, SA PRS^d^	2.6	2.7

Nagelkerke R^2^ values were derived from binary logistic regression models with self-reported suicide attempt as the dependent variable.

a Model 2 significantly explains more variance than Model 1 in the European ancestry (the likelihood ratio chi-square test, p = 0.004) but not in the African ancestry (p = 0.111).

b Model 3 significantly explains more variance than Model 1 in the European ancestry (the likelihood ratio chi-square test, p < 0.001) and in the African ancestry (p = 0.047).

c Model 4 significantly explains more variance than Model 3 in the European ancestry (the likelihood ratio chi-square test, p = 0.009) but not in the African ancestry (p = 0.096).

d PRS after standardizing the raw PRS produced by PRSice-2 at a GWAS P-value threshold of 0.05 and then regressing out the first ten within-ancestry genetic ancestry principal components.

e Suicide attempt/death. SA, suicide attempt; PRS, polygenic risk score; GWAS, Genome-wide association study.

## Discussion

We present evidence suggesting clinical utility of a polygenic score explaining suicide attempt in Black and White US youth. Two main strengths of this work are noteworthy. First, the focus on suicide attempt highlights the clinical significance of the findings. Notably, most research in this age range lumps ideation and attempt together (20, 21), even though most ideators do not make an attempt (3, 22). Second, the inclusion of Black youth in the current work is critical to address racial disparities in psychiatric genetics research (23). This disparity is especially concerning in the field of youth suicide, where Black US youth are particularly vulnerable (2, 24). Our findings extend recent ABCD Study results, showing associations of a depression polygenic risk score with suicide attempt in an analysis limited to European ancestry individuals (25) and a schizophrenia polygenic risk score with suicide attempt reported in the baseline ABCD Study assessment in admixed population with substantially fewer suicide attempt participants (64 vs. 182 in the current analysis) (26).

We found that PRS-SA additively explains variance in suicide attempt beyond parental history of suicide attempt/death. *From a clinical perspective*, assessment of family history is common practice for clinicians to help their risk classification. We believe that clinicians can intuitively appreciate the value of PRS-SA when it is compared to this benchmark of clinical good practice. *From a research perspective*, considering skepticism in the field toward incorporating PRS in multivariable predictive algorithms in psychiatry (5), our findings provide support for incorporation of genetic scores, including that of suicide attempt, in suicide risk prediction (27). We suggest that this work serves as a proof of a concept for the potential utility of integrating polygenic risk as part of the comprehensive youth suicide risk assessment. Nonetheless, it is critical to remember that etiology of suicidal behavior is complex and is driven by multiple non-genetic factors (e.g., environmental stressors, socioeconomic factors) (28, 29), which may interact among themselves (Environment by Environment interaction) (30). Additionally, factors such as preexisting psychiatric morbidity also explain substantial variance in suicide-related outcomes, including among ABCD Study participants (19).

The inclusion of African ancestry youth is a notable strength of this work. However, the trans-ancestry discovery GWAS we used (16) presented computational challenges. Such GWAS are becoming increasingly popular as a means to increase explanatory power through fine tuning and increased sample sizes (31), but they present a technical hurdle for newer Bayesian PRS computing methods. PRS-CS, for example, requires the use of an external, single-ancestry LD panel that is matched to the ancestry of a single-ancestry discovery GWAS (32). We opted to use a trans-ancestry discovery GWAS with PRSice-2 instead of using a EUR-only discovery GWAS with PRS-CS because we placed a higher priority on being able to produce PRS-SA for both African and European ancestry adolescents than on using a marginally more predictive Bayesian method that would only be feasible for computing PRS-SA for European ancestry adolescents. If an African ancestry discovery GWAS for suicide attempt had been available, we would have opted instead to use the two single-ancestry discovery GWAS to compute PRS-SA for both groups of adolescents with PRS-CS as we have done previously (20).

Our findings should be interpreted in the context of some limitations. First, the variance explained by addition of PRS-SA to models of parental history is still relatively small. Larger studies that are more high-powered with diverse samples are needed to further explore the potential of PRS-SA to explain greater variance in suicide attempts. Second, it is possible that suicide attempt was underreported by youth. Still, the ABCD Study used a well-validated tool to probe for suicide attempts. Third, we needed to exclude participants from the analyses who did not provide data on suicide attempts, on parental history, or had different genetic ancestries. Still, the included sample was diverse and included >5,000 youth. We believe that, as more longitudinal ABCD Study data become available and as more diverse GWAS becomes available, future works will be able to include more participants. Fourth, the relative size of the African ancestry population was substantially smaller than that of the European ancestry (~1,000 vs. ~4,000). Additionally, given the primarily European composition of the original GWAS, the PRS is expected to have lower predictive power in African ancestry individuals, explaining 0.69–0.88% of the phenotypic variance in suicide attempt in European ancestry populations and only 0.21–0.58% in African ancestry populations (16). This may explain the lack of statistical significance in the African ancestry-stratified models in ABCD. Nonetheless, the direction of effects was similar across ancestries, and meta-analyzed results were significant. Therefore, we believe that this work is an important step forward for the field due to the inclusion of Black youth who are at increased risk for suicide.

## Conclusions

In this cohort of young adolescents, PRS-SA was associated with suicide attempts and significantly improved models explaining variance over and above parental history of suicide attempt/death, which is commonly used in clinical settings to assess suicide risk. Findings suggest that PRS-SA may be useful for suicide risk classification in both Black and White youth.

## Data availability statement

Data used in the preparation of this article were obtained from the Adolescent Brain Cognitive Development Study (https://abcdstudy.org) held in the National Institute of Mental Health Data Archive.

## Ethics statement

All participants gave assent. Parents and/or caregivers provided written informed consent. The ABCD Study protocol was approved by the University of California, San Diego, Institutional Review Board and was exempted from a full review by the University of Pennsylvania Institutional Review Board.

## Author contributions

RB conceptualized the study question and study design, interpreted the findings, and wrote the first draft of the manuscript. EV curated and processed the phenotypic data and conducted data analysis. LS processed all genomic data, calculated the polygenic risk score, and supervised statistical analyses. VW and ND substantially helped in study conceptualization, data interpretation, and preparation of the first draft of the manuscript. LA supervised study conceptualization and all statistical analyses and contributed to data interpretation. All authors made substantial contribution to editing and revising the manuscript to its final version.

## Funding

This study was supported by the National Institute of Mental Health grants K23MH120437 (RB), R21MH123916 (RB), and the Lifespan Brain Institute of Children's Hospital of Philadelphia and Penn Medicine, University of Pennsylvania. The funding organization had no role in the design and conduct of the study; collection, management, analysis, and interpretation of the data; preparation, review, or approval of the manuscript; and decision to submit the manuscript for publication.

## Conflict of interest

RB serves on the scientific board and receives consulting fees from “Taliaz Health” and “Zynerba Pharmaceuticals” and reports stock ownership in “Taliaz Health”, with no conflict of interest relevant to this work. EV's spouse is a shareholder and executive in ‘Kidas’, with no conflict of interest relevant to this work. In the past 3 years, ND has been a consultant for Sunovion Pharmaceuticals and is on the scientific advisory board for Sentio Solutions and Circular Genomics for unrelated work. The remaining authors declare that the research was conducted in the absence of any commercial or financial relationships that could be construed as a potential conflict of interest.

## Publisher's note

All claims expressed in this article are solely those of the authors and do not necessarily represent those of their affiliated organizations, or those of the publisher, the editors and the reviewers. Any product that may be evaluated in this article, or claim that may be made by its manufacturer, is not guaranteed or endorsed by the publisher.
