# Supplementary material for: Genetic risk, parental history, and suicide attempts in a diverse sample of US adolescents
Source: Front Psychiatry. 2022 Sep 14;13:941772. doi: 10.3389/fpsyt.2022.941772 (PMC9515424; doi:10.3389/fpsyt.2022.941772)
Supplement: Supplementary file 1 [file Data_Sheet_1.PDF]

==============================

Supplemental Data

==============================

Genetic risk, parental history, and suicide attempts in a diverse sample of US adolescents

Table of Contents:

Supplemental Table 1- PRSice-2 SNP counts.

Supplemental Table 2- Determination of P-threshold for polygenic risk score of suicide attempt.

Supplemental Table 3- Ancestry stratified analyses.

Supplemental Figure 1- Sample Selection for the Current Study.

Supplemental Figure 2- Distribution of odds ratios obtained when using suicide attempt polygenic risk score at $P_{threshold}$=0.05 in 9,999 permutations.

Supplemental Table 1- PRSice-2 SNP counts

P-value threshold	SNP count after LD clumping	
	EUR	AFR
0.0001	325	416
0.001	1,527	2,024
0.01	7,332	10,772
0.05	21,216	33,589
0.1	33,279	55,202
0.3	64,214	115,614
0.5	82,451	157,628
1	102,008	214,567

Number of SNPs that entered into PRS-SA calculations after ancestry-specific LD clumping across a range of eight discovery GWAS P-values.
Abbreviations: SNP = single-nucleotide polymorphism; LD = linkage disequilibrium; EUR = European ancestry; AFR = African ancestry.

Supplemental Table 2- Determination of P-value threshold for polygenic risk score of suicide attempt in meta-analyzed model that includes African and European ancestry youth.

	Odds ratio	95% CI	P-value
Pt=1	1.2777	1.0855 – 1.5041	0.0032
Pt=0.5	1.2656	1.0747 – 1.4905	0.0048
Pt=0.3	1.2858	1.0912 – 1.5151	0.0027
Pt=0.1	1.2892	1.0946 – 1.5183	0.0023
Pt=0.05	1.3075	1.1122 – 1.5372	0.0012
Pt=0.01	1.2765	1.0881 – 1.4974	0.0027
Pt=0.001	1.241	1.0616 – 1.4507	0.0067
Pt=0.0001	1.1161	0.9503 – 1.3108	0.1808

Values were derived from meta-analyzed logistic regression models (N=5,214, including n=4,128 European-ancestry and n=1,086 African-ancestry individuals) with PRS-SA as the independent variable and suicide attempt as the dependent variable, co-varying for age and sex. Note that while we selected a GWAS p-value threshold of 0.05 for the main analyses, the association between PRS-SA and suicide attempt was broadly similar across P-value thresholds, with the exception of the most restrictive one. All PRS-SA were z-scored and had the first 10 within-ancestry genetic principal components regressed out.
Abbreviations: PRS-SA = Polygenic risk score of suicide attempt; Pt= P-value threshold.

Supplemental Table 3- Ancestry-stratified analyses for the EUR (n = 4,128) and AFR (n = 1,086) cohorts.

	Model 1: EUR			Model 1: AFR		
	OR	95%CI	p	OR	95%CI	p
Age	1.01	0.99 – 1.03	0.408	1.02	0.98 – 1.05	0.343
Female sex	0.9	0.62 – 1.31	0.588	1.59	0.98 – 2.63	0.064

	Model 2: EUR			Model 2: AFR		
	OR	95%CI	p	OR	95%CI	p
Age	1.01	0.99 – 1.03	0.391	1.02	0.98 – 1.05	0.313
Female sex	0.89	0.61 – 1.30	0.55	1.62	0.99 – 2.68	0.056
PRS-SA	1.32	1.09 – 1.60	**0.004**	1.27	0.95 – 1.72	0.113

	Model 3: EUR			Model 3: AFR		
	OR	95%CI	p	OR	95%CI	p
Age	1.01	0.99 – 1.03	0.4	1.01	0.98 – 1.05	0.364
Female sex	0.9	0.62 – 1.31	0.594	1.63	1.00 – 2.70	0.053
Parental suicide attempt/death	3.37	1.94 – 5.54	**<0.001**	2.27	1.01 – 4.58	**0.032**

	Model 4: EUR			Model 4: AFR		
	OR	95%CI	p	OR	95%CI	p
Age	1.01	0.99 – 1.04	0.375	1.02	0.98 – 1.05	0.323
Female sex	0.89	0.61 – 1.30	0.559	1.66	1.02 – 2.76	**0.045**
PRS-SA	1.29	1.07 – 1.57	**0.009**	1.29	0.96 – 1.74	0.098
Parental suicide attempt/death	3.19	1.83 – 5.27	**<0.001**	2.33	1.03 – 4.72	**0.027**

Odds ratios derived from binary logistic regression models with age, sex, suicide attempt PRS and parental history of suicide attempt/death as independent variables and self-reported suicide attempt as the dependent variable.
Model 1 includes age and sex as independent variables.
Model 2 includes age, sex, and PRS-SA as independent variables.
Model 3 includes age, sex, and suicide attempt family history as independent variables.
Model 4 includes age, sex, PRS-SA, and suicide attempt family history as independent variables.
Abbreviations: OR= odds ratio; CI= confidence interval; AFR= African ancestry; EUR= European ancestry; 95%CI= 95% confidence interval; p= P-value; PRS-SA= Polygenic risk score of suicide attempt after standardizing the raw PRS produced at a GWAS P-value threshold of 0.05 and then regressing out the first ten genetic ancestry principal components.

Supplemental Figure 1- Sample Selection for the Current Study

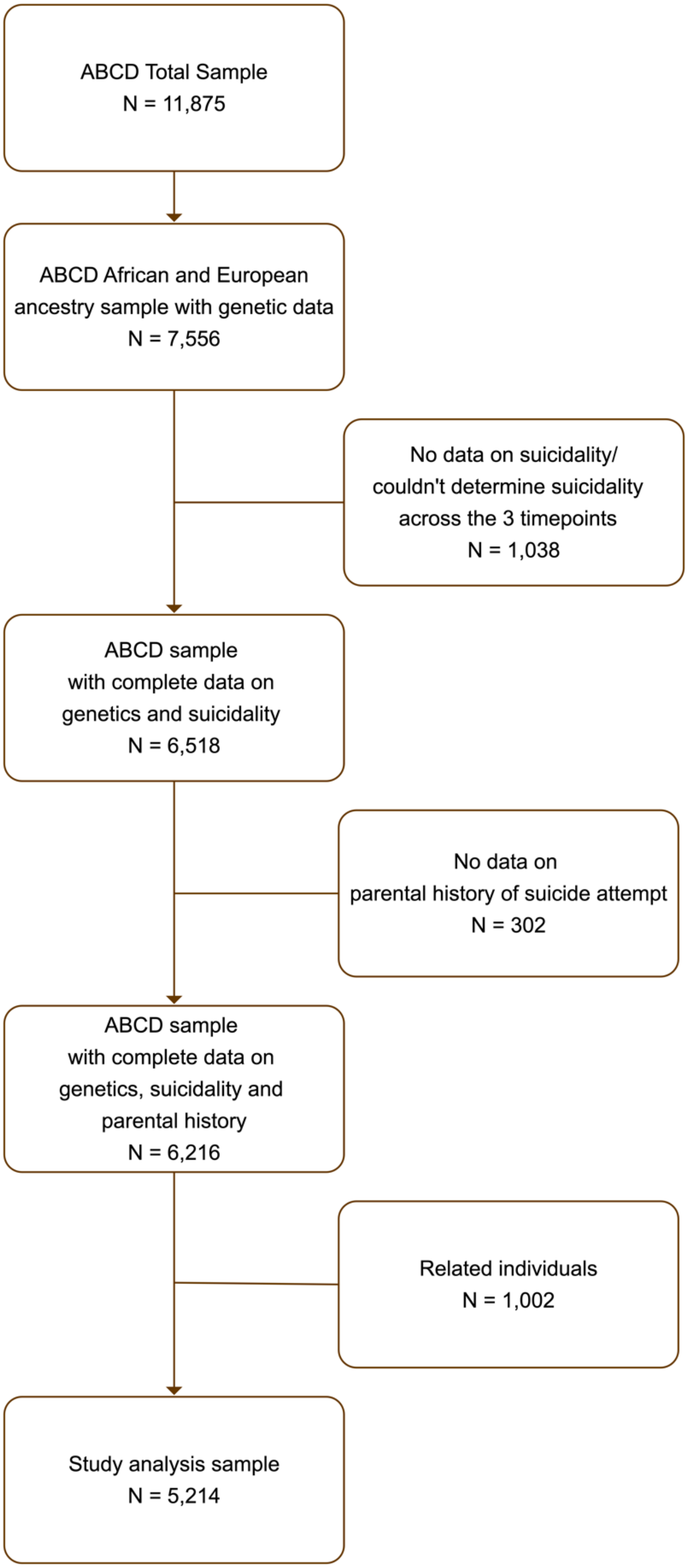

Supplemental Figure 2- Distribution of odds ratios obtained when using suicide attempt polygenic risk score at $P_{threshold}$=0.05 in 9,999 permutations.

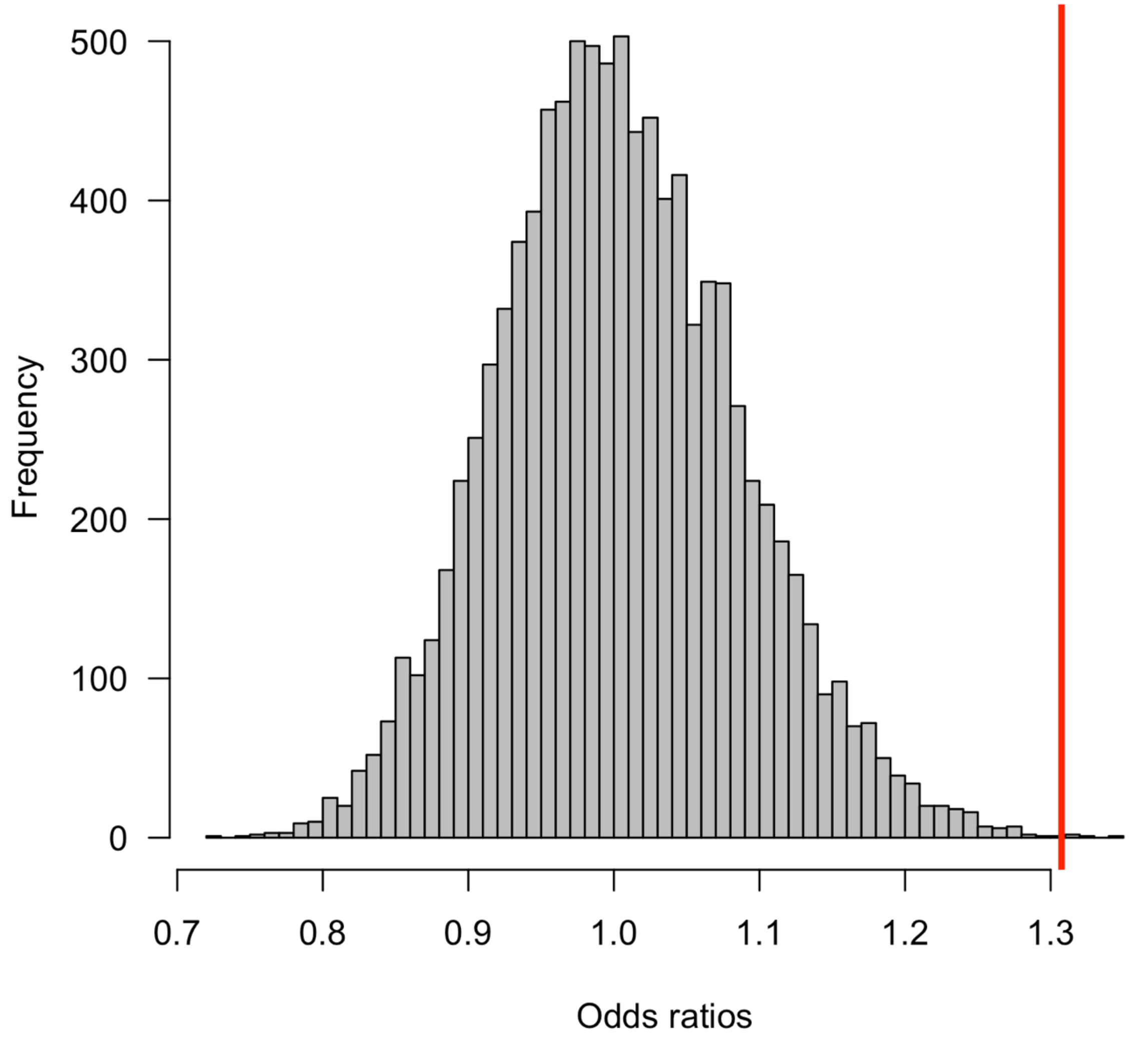

Caption: Odds ratios (x-axis) obtained with 9,999 permutations of the suicide attempt phenotype using the suicide attempt polygenic risk score (after z-scoring and regressing out ten within-ancestry genetic principal components) as the independent variable, co-varying for age and sex in the meta-analyzed models. Red line represents the location of the odds ratio obtained using the original (i.e., non-permuted) data. Only 4 out the 9,999 permutation odds ratios were greater than or equal to the original odds ratio.
